# AMPK-mediated energy homeostasis and associated metabolic effects on cancer cell response and resistance to cetuximab

**DOI:** 10.18632/oncotarget.3432

**Published:** 2015-03-20

**Authors:** Xinqun Li, Yang Lu, Haiquan Lu, Jingtao Luo, Yun Hong, Zhen Fan

**Affiliations:** ^1^ Department of Experimental Therapeutics, The University of Texas MD Anderson Cancer Center, Houston, TX, USA; ^2^ Department of Head and Neck Oncology, Tianjin Medical University Cancer Institute & Hospital, Tianjin, China; ^3^ Department of Oral Medicine, Guanghua School of Stomatology, Sun Yat-Sen University, Guangzhou, Guangdong, China

**Keywords:** EGFR, Cetuximab, HIF-1, AMPK, Cancer metabolism

## Abstract

We previously reported that cetuximab, an EGFR-blocking antibody, inhibits cancer metabolism via downregulation of HIF-1α and reverses the Warburg effect in cancer cells. Here, we report that inhibition of HIF-1 transcriptional activity by cetuximab does not necessarily lead to successful inhibition of cell proliferation. In several head and neck squamous cell carcinoma (HNSCC) cell lines, we observed a pattern of oscillating decrease and increase of intracellular ATP level after cetuximab treatment, and the magnitude and kinetics of which varied by cell line and appeared to be linked to the extent of cellular response to cetuximab. In HNSCC cells with low basal level of AMPK activity and that responded to cetuximab-induced growth inhibition, there was a transient, LKB1-dependent activation of AMPK. In contrast, HNSCC cells that had a high basal level of AMPK activity were less sensitive to cetuximab-induced growth inhibition despite effective inhibition of EGFR downstream signaling by cetuximab. Knockdown or inhibition of AMPK markedly enhanced response to cetuximab via induction of apoptosis. These findings indicate that a transient activation of AMPK is an early metabolic marker of cellular response to cetuximab and that high and sustained AMPK activity is an important mechanism by which cancer cells survive cetuximab treatment.

## INTRODUCTION

Epidermal growth factor receptor (EGFR)-mediated cell signaling is aberrantly regulated in many types of human malignancy of epithelial origin [1]. Targeting EGFR with antibodies that block binding of natural ligands to the receptor or with small-molecule compounds that specifically inhibit activation of the receptor tyrosine kinase has shown clinical activity, which led to regulatory approval of EGFR-targeted therapy for patients with metastatic cancers of the colon, head and neck, or lungs [2, 3]. However, as is true for all currently approved targeted cancer therapies, suboptimal response and even complete resistance to EGFR-targeted therapy is not uncommon in patients whose tumors have aberrant EGFR signaling [4]. The mechanisms of resistance, which includes both intrinsic and acquired resistance, are complex. The mechanisms recognized so far include mutations of key molecules downstream of EGFR, such as Ras, that render the pathways downstream of EGFR constitutively active; the presence of overlapping mechanisms that can activate pathways downstream of EGFR; and involvement of alternative pathways that drive survival and proliferation of cancer cells [5–7].

Until recently, few studies have linked response and resistance to EGFR-targeted therapy to the status of cancer cell metabolism. We believe deep understanding of this link will provide valuable insights for design of new strategies that will ultimately improve clinical impact of this promising targeted cancer therapy. It is well known that metabolism in cancer cells is reprogrammed compared with metabolism in normal cells [8–12]. To adapt to the stressful tumor microenvironment, which includes low levels of oxygen and nutrients and a high level of acidosis, cancer cells acquire many genetic and nongenetic changes that confer selective advantages in terms of not only survival but also proliferation [13]. Accumulating evidence indicates that almost every known oncogene directly or indirectly regulates targets that are connected to cancer metabolism [13]. Hypoxia-inducible factor-1 (HIF-1), a key transcription factor regulating glycolysis, plays a critical role in reprogramming cancer metabolism in favor of aerobic glycolysis (i.e., the Warburg effect), through which large amounts of biomass and reducing equivalents in the form of NADPH are generated to support unlimited proliferation of cancer cells [14, 15]. Our laboratory previously reported that cetuximab, a US Food and Drug Administration–approved EGFR-blocking antibody, downregulates the regulatory alpha subunit of HIF-1, HIF-1α [16], and that downregulation of HIF-1α is required, although may not be sufficient, for cetuximab-induced anti-proliferative effects [17]. More recently, we reported that cetuximab reverses the Warburg effect in cancer cells via inhibiting HIF-1-regulated lactate dehydrogenase A [18]. We demonstrated that cetuximab inhibits glucose consumption and lactate production and lowers intracellular ATP levels in a HIF-1α downregulation–dependent manner. Overexpression of a degradation-resistant HIF-1α mutant counteracted cetuximab-induced decline in intracellular ATP level and conferred resistance to cetuximab-induced G1-phase cell-cycle arrest [18]. These findings provide an important mechanistic link between cetuximab-induced inhibition of cell proliferation and cetuximab-induced inhibition of metabolism in targeted cancer cells.

In the current study, we expanded our study of the link between cancer cell metabolism and cancer cell response and resistance to cetuximab. Specifically, we addressed the role of AMPK [5′-adenosine monophosphate (AMP)-activated protein kinase] in cell response and resistance to cetuximab-induced inhibition of cell proliferation. AMPK is a serine/threonine kinase that is activated by upstream kinases, such as the liver kinase B1 (LKB1) tumor suppressor that integrates growth factor receptor signaling with cell energy status [19]. In response to a decline in intracellular ATP level and simultaneous increase in AMP level, the LKB1-AMPK axis is activated and quickly reprograms glucose and lipid metabolism by switching cells from active ATP consumption to active ATP production to restore cell energy balance and thereby promote cell survival [20, 21]. Thus, we hypothesized that transient activation of AMPK may serve as an early marker of cetuximab-induced inhibition of glycolysis. We further hypothesized that cancer cells with a high basal level of AMPK activity can survive cetuximab-induced inhibition of glycolysis by sustaining AMPK activation and that inhibition of AMPK can decrease cell proliferation and viability. In this study, we report findings from our studies testing these hypotheses. Our data provide novel mechanistic insights into the response and resistance of cancer cells to EGFR-targeted therapy from an energy homeostasis point of view.

## RESULTS

### Effective inhibition of the cell signaling pathways downstream of EGFR and inhibition of HIF-1 transcriptional activity by cetuximab do not necessarily lead to successful inhibition of cell proliferation

**Figure 1:**
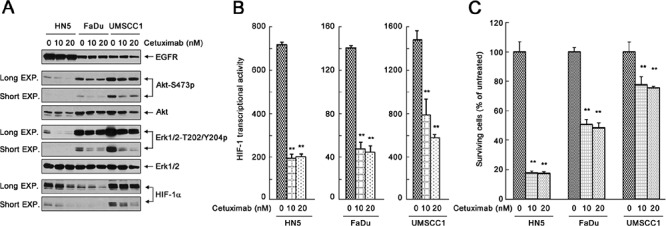
Effective inhibition of the cell signaling pathways downstream of EGFR and inhibition of HIF-1 transcriptional activity by cetuximab do not necessarily lead to successful inhibition of cell proliferation **(A)** HN5, FaDu, and UMSCC1 cells were cultured in 0.5% FBS medium in the absence or presence of 10 nM or 20 nM cetuximab for 24 h. Cell lysates were then prepared and subjected to Western blot analysis with the indicated primary antibodies. EXP, exposure. **(B)** HN5, FaDu, and UMSCC1 cells were transfected with the pBI-GL-V6L construct for 24 h. The cells were then cultured in 0.5% FBS medium in the absence or presence of 10 nM or 20 nM cetuximab in 6-well plates for 16 h. After the treatment, cell lysates were prepared for HIF-1 luciferase reporter assay. Arbitrary luciferase activity units were normalized to the amount of protein in each sample. Data shown are means and SDs (*n* = 3). *P* values for the comparisons were determined by Student's *t*-test. **p < 0.01. **(C)** HN5, FaDu, and UMSCC1 cells were cultured in 0.5% FBS medium in the absence or presence of 10 nM or 20 nM cetuximab for 5 days. The relative number of surviving cells was determined by MTT assay. The OD values of the treated groups were normalized to the OD value of untreated cells, which was set as 100%. Data shown are means and SDs (*n* = 3). **p < 0.01.

We tested our hypotheses in three human head and neck squamous cell carcinoma (HNSCC) cell lines, HN5, FaDu, and UMSCC1, which express different levels of EGFR and respond to cetuximab differentially. We first examined the changes in the levels of activation-specific phosphorylation of Akt and Erk after cetuximab by Western blot analysis (Figure 1A). Akt and Erk are well-characterized signaling molecules downstream of EGFR but can also be activated by upstream regulators other than EGFR. The basal phosphorylation levels of Akt and Erk were higher in untreated UMSCC1 cells than in untreated HN5 or FaDu cells. Consistent with the higher basal level of cell signaling in UMSCC1 cells, the basal level of HIF-1α was higher in UMSCC1 cells than in HN5 and FaDu cells (Figure 1A). The phosphorylation levels of Akt and Erk and the level of HIF-1α were all strongly inhibited upon cetuximab treatment in all three cell lines (Figure 1A). Moreover, the level of HIF-1α in these cell lines was quantitatively linked to the transcriptional activity of HIF-1 in these cells measured by a HIF-1 luciferase reporter assay, and the decline in HIF-1α level upon cetuximab treatment was accompanied by a corresponding decline in HIF-1 transcriptional activity (Figure 1B). Interestingly, however, a cell proliferation assay showed that whereas proliferation of HN5 and FaDu cells was substantially inhibited after a 5-day culture in the presence of cetuximab, proliferation of UMSCC1 cells was only minimally inhibited by cetuximab treatment; the rate of inhibition of cell proliferation was only approximately 25% in UMSCC1 cells compared to approximately 50% in FaDu cells and greater than 80% in HN5 cells (Figure 1C). These interesting findings indicate that successful inhibition of cell signaling pathways downstream of EGFR and inhibition of HIF-1 transcriptional activity by cetuximab do not always lead to successful inhibition of cell proliferation.

### Oscillation in intracellular ATP levels after cetuximab treatment in different HNSCC cell lines

**Figure 2:**
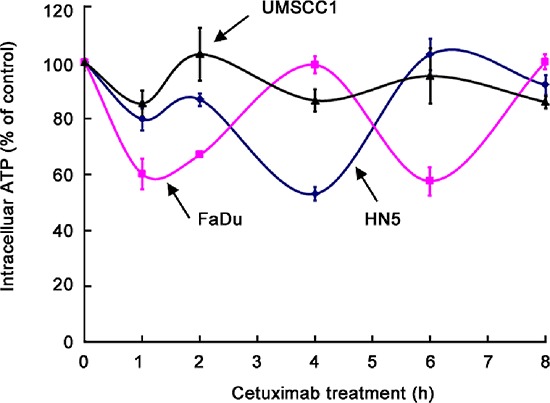
Oscillation in intracellular ATP levels after cetuximab treatment in different HNSCC cell lines HN5, FaDu, and UMSCC1 cells were cultured in medium containing 0.5% FBS and low glucose (1 g/L) with 20 nM cetuximab in triplicate wells for 1, 2, 4, 6, and 8 h. The levels of intracellular ATP were determined using the ATP Bioluminescent Assay Kit (Sigma-Aldrich). The ATP levels at each time point were normalized to the ATP levels of the untreated cells at the corresponding time points.

We recently reported that cetuximab inhibits glycolysis and lowers intracellular ATP level via downregulating HIF-1α [18]. To follow up on this work, we compared the changes in intracellular ATP levels in HN5, FaDu, and UMSCC1 cells after cetuximab treatment. During the first 8 h after addition of cetuximab in cell culture, during which time there was no detectable change in cell number (data not shown), we observed the following interesting findings with respect to intracellular ATP level (Figure 2). First, upon exposure to cetuximab, the level of intracellular ATP declined in all three cell lines. However, the ATP level did not keep declining as one would expect; rather, in all three cell lines, after an initial decline, the level of intracellular ATP reverted to a level close to the baseline level. Then, the level of intracellular ATP dropped again and then reverted again, in an oscillating pattern. Second, the kinetics of the oscillation in intracellular ATP level after cetuximab treatment differed by cell line. The period of oscillation was longer in FaDu cells (4 h) than in HN5 and UMSCC1 cells (2 h). Third, the magnitude of decline in ATP level in each oscillation cycle differed by cell line, with FaDu cells showing the sharpest decline (40% decline within 1 h) and UMSCC1 cells showing only modest decline (15% decline within 1 h).

The findings that all three cell lines displayed oscillating cycles of decline and increase in ATP level and that the decline in intracellular ATP level in UMSCC1 cells was modest compared with the decline in ATP level in the other two cell lines, which were much more sensitive to cetuximab, suggest that the magnitude of decline in intracellular ATP level and the kinetics of intracellular ATP-level oscillation may be related to cellular response to cetuximab-induced growth inhibition. Our findings further suggest that the minimal response of UMSCC1 cells to cetuximab-induced growth inhibition, compared to the response of HN5 and FaDu cells, might be due to ability of UMSCC1 cells to limit the extent of decline in ATP level after cetuximab treatment.

### Transient activation of AMPK after cetuximab treatment in different HNSCC cell lines

AMPK is a key metabolic sensor that helps maintain cellular energy homeostasis in response to a declining level of intracellular ATP and a simultaneously rising AMP level [20, 21]. We thus first examined whether AMPK was activated after cetuximab treatment in HN5, FaDu, and UMSCC1 cells.

**Figure 3:**
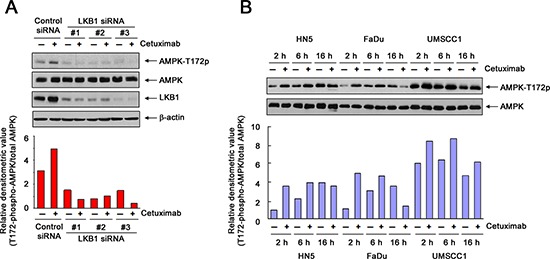
Transient activation of AMPK after cetuximab treatment in different HNSCC cell lines **(A)** HN5 cells were transfected with a control siRNA or one of three different LKB1-targeting siRNAs (designated as #1, #2, and #3, respectively). Forty-eight hours after transfection, the cells were treated with 20 nM cetuximab or not for 1 h. Cell lysates were then prepared and subjected to Western blot analysis with the indicated primary antibodies. The densitometric value of T172-phosphorylated AMPK in each lane was normalized to the densitometric value of total AMPK in the corresponding lane and plotted in arbitrary units. **(B)** HN5, FaDu, and UMSCC1 cells were cultured in 0.5% FBS medium in the absence or presence of 10 nM or 20 nM cetuximab for 2, 6, and 16 h. Cell samples were subjected to Western blot analysis for levels of T172-phosphorylated AMPK and total AMPK, and the densitometric value of T172-phosphorylated AMPK in each sample was analyzed as described in (A).

By using Western blot analysis to detect changes in the level of AMPK activation-specific phosphorylation on threonine 172 (AMPK-T172), we observed an increase in AMPK activity in HN5 cells after 4-h exposure to cetuximab (Figure 3A). The increase in AMPK activity after cetuximab treatment was abolished when the expression of LKB1 was silenced by any one of three LKB1 siRNAs, indicating that cetuximab activated AMPK via LKB1, i.e., cetuximab exerted this effect through the canonical LKB1-AMPK axis.

We next compared the changes in AMPK activity over an extended period after cetuximab treatment in all three HNSCC cell lines. We found that the level of AMPK-T172 relative to the level in untreated cells was increased in all three cell lines as early as 2 h after cetuximab treatment (Figure 3B). It is noteworthy that when the cells were cultured in serum-starved medium overnight (16 h), the level of AMPK-T172 also increased in untreated cells, which reflects the consumption and gradual depletion of nutrients in the medium. In HN5 and FaDu cells, after 2 h, there was no further increase (and there was even a decrease in FaDu cells) in the AMPK-T172 level in cetuximab-treated cells compared to the level in the corresponding untreated cells. In contrast, in UMSCC1 cells, up to 16 h, the level of AMPK-T172 remained higher in cetuximab-treated cells than in the corresponding untreated cells.

These findings suggest two conclusions: first, following cetuximab treatment, there is initially a transient activation of AMPK to counteract and allow cells to recover from the cetuximab-induced decline in ATP; this transient activation of AMPK may explain the oscillation in the level of intracellular ATP after cetuximab treatment and may also serve as a marker of positive response to cetuximab treatment. Second, the high basal level of activated AMPK (AMPK-T172) in UMSCC1 cells and the persistent high level of AMPK-T172 after extended cetuximab treatment may account for the minimal inhibitory effect of cetuximab on proliferation of UMSCC1 cells, compared to the inhibitory effect on HN5 and FaDu cells.

### Transient activation of AMPK counteracts cetuximab-induced decline in intracellular ATP level

To confirm the role of AMPK activity in maintaining intracellular ATP level after cetuximab treatment, we examined the effect of cetuximab on intracellular ATP level in HN5 and UMSCC1 cells with and without AMPK silencing.

**Figure 4:**
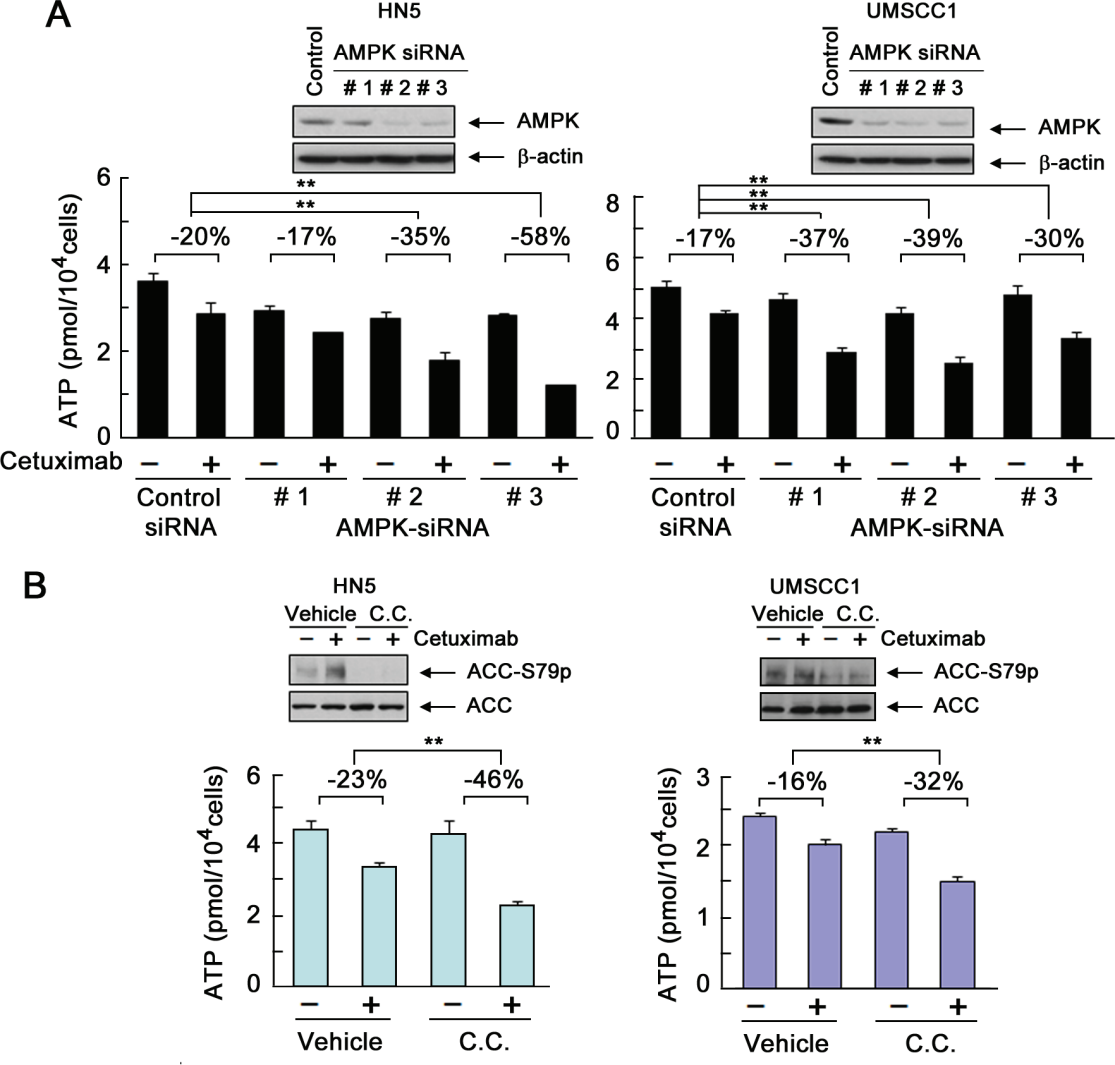
Transient activation of AMPK counteracts cetuximab-induced decline in intracellular ATP level **(A)** HN5 and UMSCC1 cells were transfected with a control siRNA or one of three different AMPK-targeting siRNAs (designated as #1, #2, and #3, respectively). Forty-eight hours after transfection, the cells were treated with 20 nM cetuximab or not for 1 h. Intracellular levels of ATP in each sample were measured using the ATP Bioluminescent Assay Kit (Sigma-Aldrich). The negative percentages indicate the extent of inhibition of intracellular ATP level by cetuximab compared with the ATP level in the control group under each of the indicated conditions. **p < 0.01. Insets, Western blots showing knockdown of AMPK expression in cells transfected with AMPK-targeting siRNAs or control siRNA. **(B)** HN5 and UMSCC1 cells were cultured in 0.5% FBS medium in the presence of 10 μM dorsomorphin (C.C. for “compound C”) for 16 h and then treated with 20 nM cetuximab or not for 1 h. Cell samples were prepared for measurement of intracellular ATP level, and the data were expressed as described in (A). Insets, Western blots showing inhibition of S79 phosphorylation of ACC by dorsomorphin.

As shown by Western blotting, two of three AMPK siRNAs that we tested successfully silenced AMPK in HN5 cells (Figure 4A, left panel, inset). These AMPK-silenced HN5 cells were used to assess the impact of AMPK silencing on cetuximab-induced decline in intracellular ATP level. In HN5 cells, a 1-h exposure to cetuximab led to a modest 20% decline in intracellular ATP level. Knockdown of AMPK alone also modestly lowered the intracellular ATP level, which was expected because of the role of AMPK in maintaining total intracellular ATP level. Importantly, knockdown of AMPK led to greater declines in intracellular ATP level after cetuximab treatment. Consistent with the differences in their efficiency in silencing AMPK, siRNA#1 weakly enhanced the decline in intracellular ATP level after cetuximab treatment (enhanced by 17%), but siRNA#2 and siRNA#3 substantially enhanced the decline in intracellular ATP level after cetuximab treatment (enhanced by 35% and 58%, respectively) (Figure 4A, left panel). Similar findings were seen in UMSCC1 cells, which had a higher basal level of AMPK as shown by Western blotting (Figure 4A, right panel, inset). In UMSCC1 cells, each of the three AMPK siRNAs successfully silenced AMPK. We found that the modest decline in intracellular ATP level in UMSCC1 cells after cetuximab was significantly enhanced when the expression of AMPK was knocked down; the decline in ATP level after cetuximab treatment was 17% in control siRNA-treated cells but 37%, 39%, and 30%, respectively, in the three different AMPK siRNA-treated cells.

To further confirm that our findings were a direct effect of inhibition of AMPK activity, we treated HN5 and UMSCC1 cells with dorsomorphin (also known as compound C or C.C.), a well-characterized and widely used small-molecule kinase inhibitor of AMPK [22]. Figure 4B shows that pre-exposure of HN5 and UMSCC1 cells to dorsomorphin led to a lower AMPK activity than was observed in vehicle-treated control cells, indicated by Western blotting findings (insets) of decreased level of phosphorylation of acetyl CoA carboxylase (ACC) on serine 79 (ACC-S79), a site known to be specifically phosphorylated by AMPK [23]. In both HN5 and UMSCC1 cells, the decline in intracellular ATP level after cetuximab treatment was greater in the cells pre-exposed to dorsomorphin than in the cells pre-exposed to vehicle control. We found that the impact of AMPK knockdown (Figure 4A) or kinase inhibition (Figure 4B) on cetuximab-induced decline in intracellular ATP level was greater in HN5 cells, which are very sensitive to cetuximab, than in UMSCC1 cells, which are only minimally responsive to cetuximab-induced growth inhibition (Figure 1C). UMSCC1 cells are hereinafter referred to as “cetuximab-resistant cells” to distinguish them from cetuximab-sensitive HN5 cells.

These findings, taken together with the knowledge that AMPK is a metabolic sensor that responds to energy deficiency by switching cells from active ATP consumption to active ATP production, confirm that transient activation of AMPK after cetuximab treatment counterbalanced cetuximab-induced decline in intracellular ATP level and thereby restored the ATP level in the targeted cells.

### Inhibition of AMPK activation enhances responses of cetuximab-resistant cells to cetuximab

**Figure 5:**
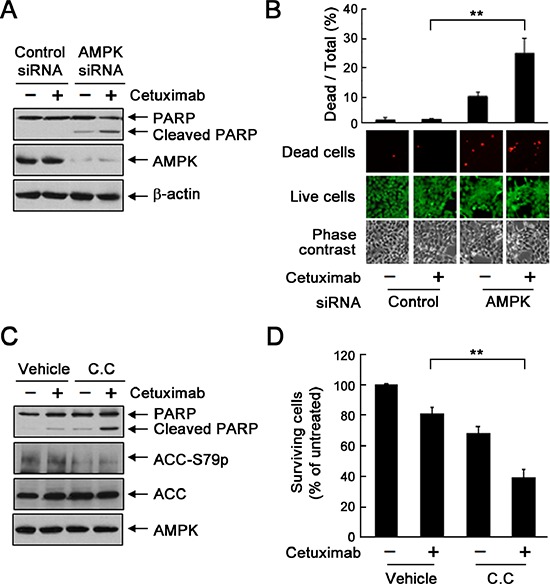
Knockdown or inhibition of AMPK sensitizes UMSCC1 cells to cetuximab treatment via induction of apoptosis **(A)** and **(B)** UMSCC1 cells were subjected to knockdown of AMPK with siRNA #2. Forty-eight hours after transfection, the cells were treated with 20 nM cetuximab or not for 24 h. In (A), cell lysates were prepared and subjected to Western blot analysis with the indicated primary antibodies. In (B), the cells were subjected to a LIVE/DEAD assay (Invitrogen) and observed under a fluorescent microscope. The ratio of dead cells to total cells in each group was calculated as described in Materials and Methods. **p < 0.01. **(C)** UMSCC1 cells were cultured in 0.5% FBS medium in the presence of 20 nM cetuximab, 10 μM dorsomorphin (C.C. for “compound C”), or both for 24 h. Cell lysates were then prepared and subjected to Western blot analysis with the indicated primary antibodies. **(D)** UMSCC1 cells were treated with 20 nM cetuximab alone or in combination with 2.5 μM dorsomorphin for 72 h. Cell viability was determined with MTT assay. The OD values of the treated cells were normalized to the OD value of the untreated cells, which was set as 100%. Data shown are means and SDs (*n* = 3). **p < 0.01.

To determine whether inhibition of AMPK activation enhances the response of cetuximab-resistant UMSCC1 cells to cetuximab, we treated the cells with cetuximab with and without knockdown of AMPK by siRNA or inhibition of AMPK with dorsomorphin. Figure 5A shows that knockdown of AMPK led to induction of apoptosis, shown by cleavage of the nuclear protein poly(adenosine diphosphate ribose) polymerase (PARP), a marker of apoptosis, after cetuximab treatment. The induction of apoptosis was further confirmed by cell LIVE/DEAD assay showing only 1.3% of cells dead upon cetuximab treatment and 9.3% of cells dead upon AMPK knockdown but 24.7% of cells dead with the combination (Figure 5B). Similar findings were observed with dorsomorphin treatment (Figure 5C). An MTT assay showed that the combination of cetuximab and dorsomorphin resulted in significantly greater growth inhibition or cell killing than either treatment alone (Figure 5D).

**Figure 6:**
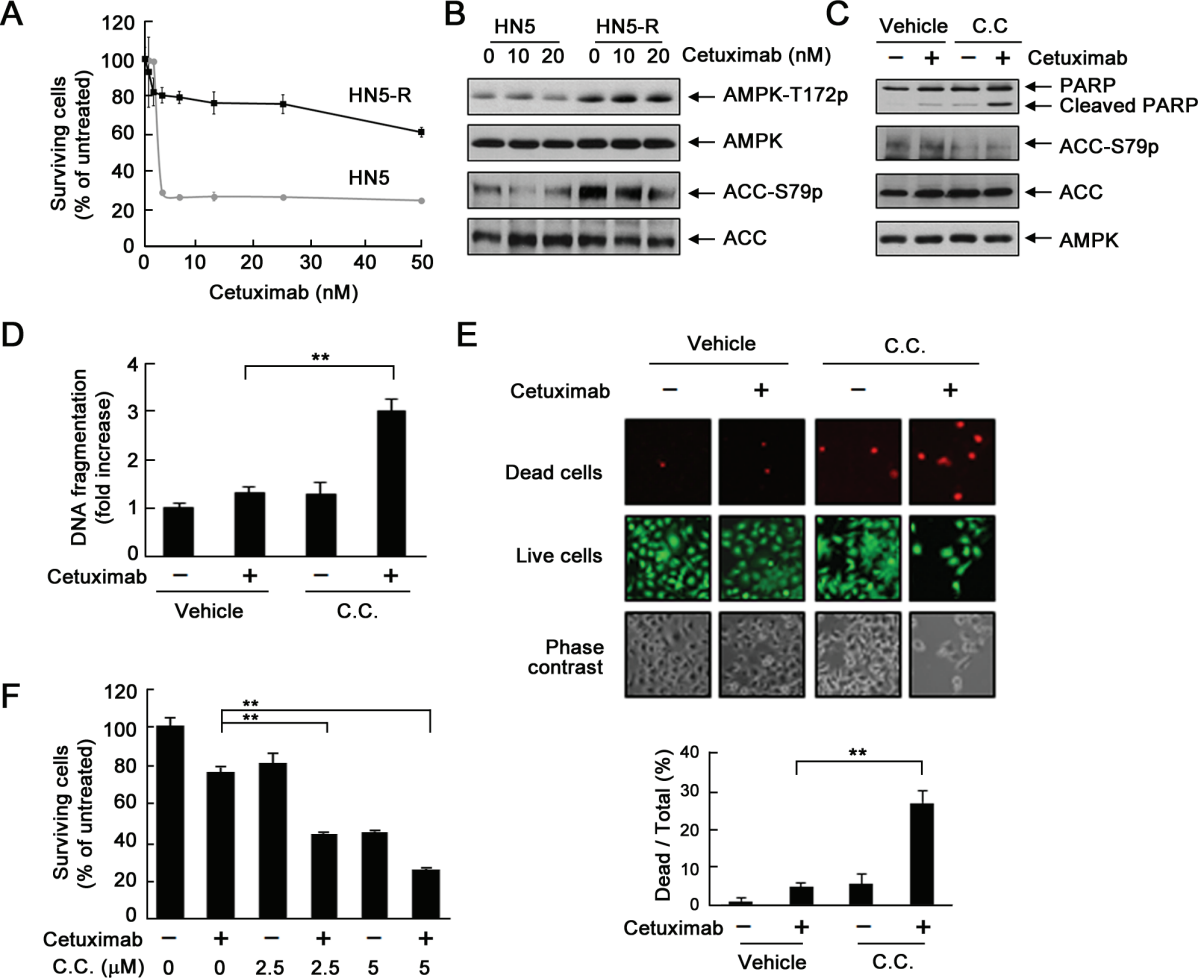
Inhibition of AMPK overcomes acquired resistance to cetuximab via induction of apoptosis **(A)** HN5 cells and HN5-R cells were cultured in 0.5% FBS medium containing the indicated concentrations of cetuximab for 5 days and then subjected to MTT assay. **(B)** HN5 and HN5-R cells were cultured in 0.5% FBS medium in the absence or presence of 10 or 20 nM cetuximab for 24 h. Cell lysates were then prepared and subjected to Western blot analysis with the indicated primary antibodies. **(C)** HN5-R cells were cultured in 0.5% FBS medium in the presence of 20 nM cetuximab, 10 μM dorsomorphin (C.C. for “compound C”), or both for 24 h. Cell lysates were then prepared and subjected to Western blot analysis with the indicated primary antibodies. **(D)** HN5-R cells were treated as described in (C). Cell lysates were subjected to a quantitative apoptosis ELISA (Roche Diagnostics). **(E)** HN5-R cells were treated as described in (C). The cells were subjected to a LIVE/DEAD assay (Life Technologies) and observed under a fluorescent microscope. The ratio of dead cells to total cells in each group was calculated as described in Materials and Methods. **p < 0.01. **(F)** HN5-R cells were treated with 20 nM cetuximab alone or in combination with 2.5 or 5 μM dorsomorphin for 72 h. Cell viability was determined by MTT assay. The OD values of the treated cells were normalized to the OD value of the untreated cells, which was set as 100%. Data shown are means and SDs (*n* = 3). **p < 0.01.

To further confirm a role of AMPK in development of acquired resistance to cetuximab, we exposed parental HN5 cells to cetuximab continuously for over 6 months with stepwise increases in cetuximab concentration in culture medium from 0.2 nM to 20 nM [18], which led to development of a cetuximab-resistant HN5 subline (HN5-R). The response of HN5-R cells to cetuximab treatment was similar to the response of UMSCC1 cells (Figure 1C): HN5-R cells exhibited only a modest, 20–25% inhibition by cetuximab at 50 nM, compared to an over 75% inhibition by cetuximab at 2.5 nM in parental HN5 cells (Figure 6A). Compared with parental HN5 cells, HN5-R cells had higher basal AMPK activity, shown by higher levels of T172-phosphorylated AMPK and S79-phosphorylated ACC (Figure 6B). Similar to findings in UMSCC1 cells (Figure 5), treatment of HN5-R cells with cetuximab plus dorsomorphin resulted in apoptosis as indicated by Western blotting showing increased cleavage of PARP (Figure 6C), as well as quantitative enzyme-linked immunosorbent assay (ELISA) showing increased DNA fragmentation (Figure 6D). In contrast, treatment with cetuximab or dorsomorphin alone had no obvious effects (Figure 6C and 6D). We also conducted additional assays to ascertain the cell-killing effect of the combination treatment at the cell level. Both the LIVE/DEAD cell viability assay and MTT assay showed significantly greater cell death with combination treatment than with either treatment alone (Figure 6E and 6F).

To confirm the reproducibility of these findings in cancer types other than head and neck cancer, we examined the effect of the combination of cetuximab and dorsomorphin in a comparable cell line derived from colorectal cancer, the other cancer type for which cetuximab is approved [24–26]. DiFi5 is a subline with acquired resistance to cetuximab generated from DiFi colorectal cancer cells [27]. We found that treatment of DiFi5 cells with the combination of cetuximab and dorsomorphin produced effects on PARP cleavage and cell death similar to the effects observed in HN5-R cells (Supplementary Figure S1).

## DISCUSSION

In this study, we gained new mechanistic perspective on the response and resistance of cancer cells to cetuximab treatment from a standpoint of AMPK-mediated energy homeostasis and associated metabolic effects. This study is a follow-up to our recent work reporting that cetuximab exerts its antitumor activity in part via reversing the Warburg effect in cancer cells [18] and our earlier work reporting that cetuximab can downregulate HIF-1α and that downregulation of HIF-1α is required for cetuximab-induced antitumor activities [16, 17]. Our findings from both biochemical and cell-based assays support the conclusions that a transient activation of AMPK is an early metabolic marker of response to cetuximab-induced inhibition of glycolysis, and high and sustained AMPK activity helps cancer cells survive under the stress of cetuximab-induced inhibition of cancer metabolism.

Successful inhibition of the signaling pathways downstream of EGFR is sometimes equated with the anti-proliferative and anti-survival effects of EGFR-targeted therapy because it is required for successful inhibition of cell proliferation and, under certain circumstance, induction of cell death by EGFR-targeted therapy. Our current data indicate, however, that while successful inhibition of the downstream pathways is required, it may not necessarily lead to successful inhibition of cell proliferation or induction of cell death. This concept shift is based on our novel findings from the current study that 1) there was an oscillating pattern of decline and increase in the level of intracellular ATP in targeted cancer cells after exposure to cetuximab, 2) the magnitude of response to cetuximab-induced decline of intracellular ATP level was linked to response to cetuximab-induced growth inhibition, 3) knockdown or inhibition of AMPK enhanced cetuximab-induced decline of intracellular ATP level, and 4) knockdown or inhibition of AMPK enhanced cetuximab-mediated effects by inducing apoptosis.

AMPK is typically activated in response to reduced cell bioenergetic metabolism in order to maintain energy homeostasis by stimulating fatty acid oxidation [28, 29] and suppressing cell biosynthesis through inhibition of the mTOR pathway and lipogenic pathway [30, 31]. Activation of AMPK occurs when the glucose supply is reduced or when glucose metabolism is inhibited. In that regard, a transient activation of AMPK in cetuximab-targeted cancer cells can be interpreted as a metabolic marker of cetuximab-induced inhibition of glycolysis. By demonstrating that knockdown or inhibition of AMPK resulted in a greater decline of intracellular ATP after cetuximab treatment, we confirmed that AMPK-mediated energy homeostasis can act as a cushion to counterbalance cetuximab-induced decline in intracellular ATP level.

The heterogeneity of cancer metabolism has been increasingly recognized in recent years [32, 33]. We found that cancer cells with a high basal level of AMPK activity (e.g., UMSCC1 cells) were resistant or minimally responsive to cetuximab-induced growth inhibition despite effective inhibition of cell signaling pathways downstream of EGFR. Furthermore, compared to parental cetuximab-sensitive cells (e.g., HN5 cells), cells that acquired resistance to cetuximab (e.g., HN5-R) exhibited an increased basal level of AMPK. By demonstrating that knockdown or inhibition of AMPK enhanced cetuximab-mediated effects by inducing apoptosis, we confirmed that a high level of AMPK protects cells from cetuximab-induced effects on cancer metabolism.

However, the role of AMPK observed in our study may not be limited to maintaining energy homeostasis after cetuximab treatment. In the current study, we did not explore in depth the mechanisms by which AMPK influences cancer cell response to cetuximab. We speculate that cancer cells that are naturally resistant to cetuximab or have acquired resistance to cetuximab may use parallel or alternative metabolic pathways, such as via increased glutaminolysis, to various degrees, to obtain needed energy and biomass. Glutaminolysis may provide alternative energy and biomass to support cell survival and proliferation when glycolysis is inhibited by cetuximab. Because glutaminolysis takes place partly in the mitochondria, excess glutaminolysis could lead to overproduction of reactive oxygen species (ROS). While normally the level of ROS is limited by cellular antioxidant systems and thereby cells are protected from ROS-induced damage, excessive production of ROS as a result of reprogrammed cancer metabolism could damage cells. AMPK may help maintain homeostasis of intracellular redox after cetuximab treatment. AMPK is well known to jump-start autophagy directly via phosphorylation of Ulk1/ATG1 [34–36] and indirectly via inactivation of mTOR through phosphorylation of TSC2 [30] or Raptor [31]. It is interesting that the level of activated AMPK was clearly increased in cetuximab-resistant cells (such as HN5-R and DiFi5). We found the activity of Src was significantly higher in HN5-R cells than in HN5 cells (data not shown) and significantly higher in DiFi5 cells than in DiFi cells [27]. AMPK thus could also be activated by Src activity through LKB1 independently of the AMP/ATP ratio [37–40]. Through regulating autophagy, AMPK maintains mitochondrial integrity in cancer cells. We previously reported that autophagy occurs after cetuximab treatment and that knockdown of autophagy regulatory genes, such as Beclin 1 or Atg7, or treatment with the lysosome inhibitor chloroquine sensitized cancer cells to cetuximab-induced apoptosis [41, 42]. We thus speculate that AMPK may also regulate autophagy to maintain mitochondria integrity when the cancer metabolism is pushed partially or completely away from glycolysis to alternative metabolic pathways in mitochondria. Under such a scenario, inhibition or knockdown of AMPK would disable the cell's ability to maintain redox homeostasis, leading to a catastrophic outcome through apoptosis. We are currently testing this hypothesis.

Separately, it is noteworthy that the transient or sustained activation of AMPK to maintain energy hemostasis after cetuximab-induced inhibition of glycolysis is mechanistically different from activation of AMPK following inhibition of the respiratory chain in mitochondria by complex I inhibitors, such as phenformin and metformin, which results in depletion of intracellular ATP and energy crisis in the treated cells and which has been reported to cause cancer cell death rather than help cancer cells survive [43–46].

In summary, our data suggest that cetuximab inhibits cancer cell proliferation through inhibition of glycolysis and that cancer cells respond to the inhibition of glycolysis by reprogramming of cell metabolism and activation of AMPK. Sustained high AMPK activity helps cancer cells survive cetuximab-induced inhibition of cancer metabolism. The remarkable variation among cancer patients in response to EGFR-targeted therapy remains a daunting challenge. Improved understanding of the ability of cancer cells to sense energy deficiency and restore energy homeostasis will help us meet that challenge by designing new strategies for improved clinical outcomes.

## MATERIALS AND METHODS

### Reagents

Cetuximab (ImClone Systems, an Eli Lilly company, New York, NY) was purchased from the pharmacy of MD Anderson Cancer Center. All other reagents were purchased from Sigma-Aldrich (St. Louis, MO) unless otherwise specified.

### Cell lines and cell cultures

HN5, FaDu, and UMSCC1 HNSCC cells and DiFi colorectal cancer cells were maintained in Dulbecco's modified Eagle's medium/F12 medium supplemented with 10% fetal bovine serum (FBS), 2 mM glutamine, 100 U/mL penicillin, and 100 μg/mL streptomycin in a 5% CO_2_ atmosphere at 37°C as described previously [47, 48]. Cetuximab-resistant HN5-R and DiFi5 cells were generated by exposing parental HN5 and DiFi cells to stepwise increasing doses of cetuximab (up to 20 nM) over 6 months or more [18, 27].

### Cell proliferation assay

Cells were cultured in 24-well plates in a 5% CO_2_ atmosphere at 37°C. Following the indicated treatments, 10 mg/mL methylthiazolyldiphenyl-tetrazolium bromide (MTT) was added (50 μL/well), and the cells were incubated for an additional 2 h. The cells were then lysed with a lysis buffer (500 μL/well) containing 20% sodium dodecyl sulfate in dimethyl formamide/H_2_O (1:1, v/v; pH 4.7) at 37°C for at least 6 h. The relative number of surviving cells in each group was determined by measuring the optical density (OD) of the cell lysates at an absorbance wavelength of 570 nm. The OD value of each treatment group was expressed as a percentage of the OD value of the untreated control cells [18].

### Knockdown of LKB1 or AMPK gene expression by siRNA

LKB1–targeted siRNAs (siRNA sequence #1, GUACUUCUGUCAGCUGAUU; #2, CGGAGAAGCG UUUCCCAGU; #3, GCUCUUACGGCAAGGU GAA), AMPK–targeted siRNAs (siRNA sequence #1, CCCAUAUUAUUUGCGUGUA; #2, CAAAUG CUUCCAUUUGUAA; #3 CAAAGUCGACC AAAUGAUA), and control siRNA were purchased from Sigma-Aldrich. The siRNA (200 pmol) and Lipofectamine 2000 (5 μL; Life Technologies) were mixed in 100 μL of minimal essential medium (Opti-MEM, Life Technologies) for 15 min, and the siRNA/Lipofectamine 2000 mixture was added into the culture medium. Six hours later, the medium was replaced with regular medium, and the cells were cultured for an additional 48 h prior to the detection of knockdown of LKB1 or AMPK expression by Western blotting.

### Western blot analysis

After the indicated treatments, cells were lysed in a lysis buffer containing 50 mM TrisHCl (pH 7.4), 150 mM NaCl, 0.5% Igepal CA-630, 50 mM NaF, 1 mM Na_3_VO_4_, 1 mM phenylmethylsulfonyl fluoride, 25 μg/mL aprotinin, and 25 μg/mL leupeptin and kept on ice for 15 min. The lysates were cleared by centrifugation, and the supernatants were collected. Equal amounts of protein lysate, as determined using the Pierce Coomassie Plus colorimetric protein assay (Thermo Fisher Scientific), were separated by sodium dodecyl sulfate–polyacrylamide gel electrophoresis, blotted onto nitrocellulose, and probed with various primary antibodies, which were all commercially available from the following vendors: Cell Signaling Technology (for antibodies against Akt, S473-phosphorylated Akt, T202/Y204-phosphorylated Erk, LKB1, AMPK, T172-phosphorylated AMPK, S79-phosphorylated ACC, and PARP), Santa Cruz Biotechnology (for antibodies against Erk and ACC), BD Biosciences (for antibody against HIF-1α), and Sigma-Aldrich (for antibodies against EGFR and β-actin). The signals were visualized using the enhanced chemiluminescence detection kit (GE Healthcare). Western blotting results were quantified using the ImageJ v1.46 image processing program (National Institutes of Health, Bethesda, MD).

### HIF-1 luciferase reporter assay

The HIF-1-luciferase reporter gene construct pBI-GL-V6L contains six tandem repeats of the hypoxia response element from the human VEGF gene [49]. HN5, FaDu, and UMSCC1 cells were transiently transfected with the pBI-GL-V6L construct using Lipofectamine 2000. After a 24-h transfection period, the cells were washed twice with phosphate-buffered saline and cultured for an additional 16 h in 0.5% FBS medium in the presence or absence of 20 nM cetuximab. The cells were then harvested and lysed in a lysis buffer (0.2 M Tris HCl [pH 8.0] and 0.1% Triton X-100). The luciferase assay was performed by adding luciferase substrate solution (0.5 mM D-luciferin, 0.25 mM coenzyme A, 20 mM Tris HCl, 4 mM MgSO_4_, 0.1 mM EDTA, 30 mM DTT, and 0.5 mM ATP) to the samples and immediately measuring for luciferase activity using a FLUOstar Omega luminometer. Arbitrary luciferase activity units were normalized to the amount of protein in each sample. The protein concentration was determined using the Pierce BCA protein assay kit.

### Intracellular ATP assay

Intracellular ATP levels were determined using the ATP Bioluminescent Assay Kit (Sigma-Aldrich) as we previously described [18]. Briefly, cells were seeded in 6-well plates at 5 × 10^5^ cells/well and treated with 20 nM cetuximab or left untreated in low-glucose, low-serum medium for 4 h. The cells were then harvested and resuspended in 1 mL of phosphate-buffered saline. An aliquot of 50 μL of the cell suspension was mixed with 100 μL of ATP-releasing reagent and 50 μL of distilled water in each well of a 96-well plate. The samples (100 μL) in each well were then transferred to a white opaque 96-well plate whose wells were each pre-filled with 100 μL of ATP assay mix. The amount of light emitted in each well was immediately measured using a FLUOstar Omega luminometer.

### Apoptosis assays

Apoptosis was measured using a colorimetric ELISA kit (Cell Death Detection ELISA, Roche Diagnostics Corp.) that quantitatively measures cytoplasmic histone-associated DNA fragments (mononucleosomes and oligonucleosomes). The procedure was performed exactly according to the manufacturer's instructions. Apoptosis was also detected by Western blotting with an antibody that recognizes both cleaved and uncleaved PARP (Cell Signaling Technology) as described previously [17, 50].

### LIVE/DEAD cell viability assay

The fluorescence-based LIVE/DEAD cell viability assay kit was purchased from Life Technologies. Following treatments, cells were incubated with 4 μM calcein acetoxymethyl ester (calcein AM) and 2 μM ethidium homodimer-1 (EthD) together in a 37°C, 5% CO_2_ incubator for 45 min. The cells were then rinsed gently with phosphate-buffered saline and then observed for cell viability under a fluorescence microscope. Live cells were identified by a green fluorescence that was produced when the non-fluorescent calcein AM was converted to calcein, which fluoresces when excited at 485 nm; dead cells were identified by a bright red fluorescence that was produced by EthD excited at 544 nm.

For quantification of the ratio of live to dead cells, cells were seeded in a clear-bottom, opaque-wall 96-well plate. After indicated treatments, cells stained with calcein AM and EthD as described above were analyzed with a fluorescence microplate reader with emission wavelength at 610 nm for EthD and at 520 nm for calcein. The relative values of live and dead cells in the treated groups were expressed as a percentage of the fluorescence reading of the corresponding group of untreated cells.

### Statistical analysis

Student's *t*-test was used for all statistical analyses. The data are presented as means and standard deviations (SDs). All experiments were repeated at least once with reproducible findings.

## SUPPLEMENTARY FIGURE


