# Supplementary material for: AMPK-mediated energy homeostasis and associated metabolic effects on cancer cell response and resistance to cetuximab
Source: Oncotarget. 2015 Mar 20;6(13):11507–18. doi: 10.18632/oncotarget.3432 (PMC4484472; doi:10.18632/oncotarget.3432)
Supplement: Supplementary file 1 [file oncotarget-06-11507-s001.pdf]

SUPPLEMENTARY FIGURE

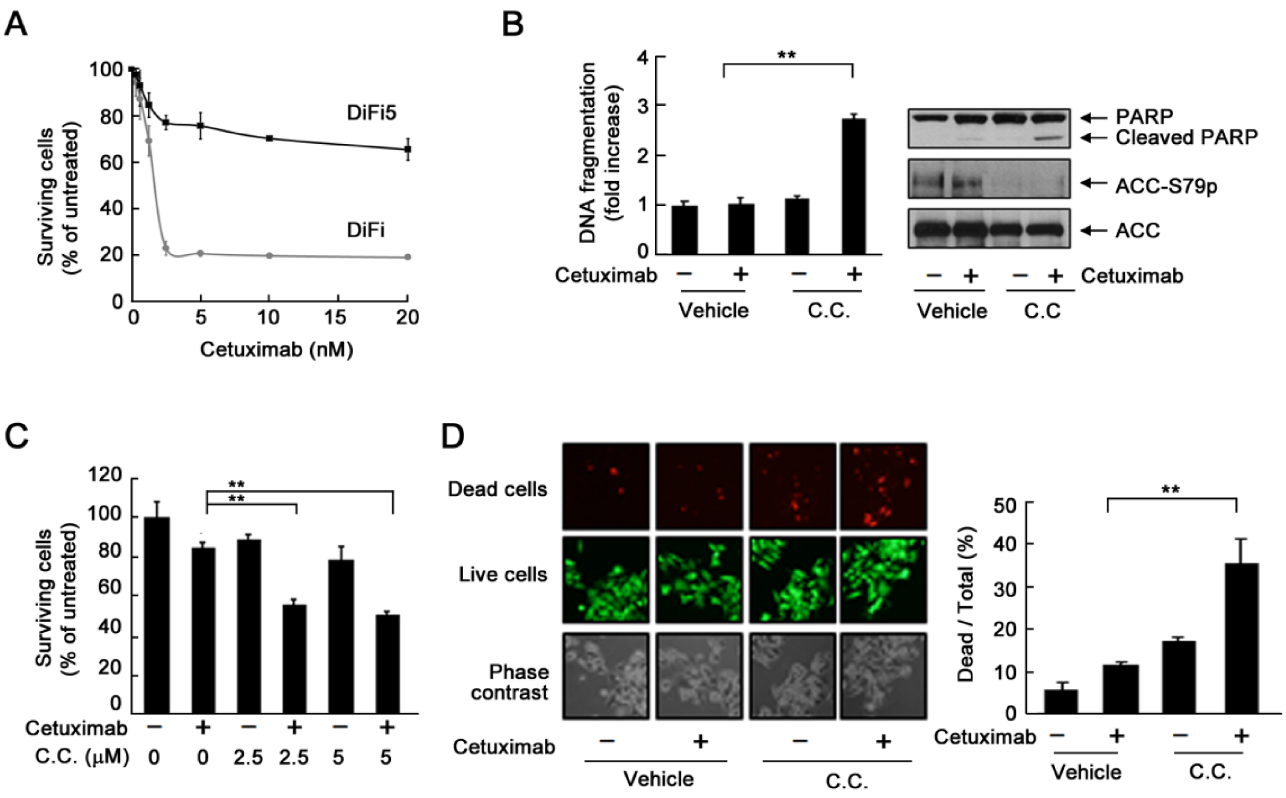

Supplementary Figure S1: Inhibition of AMPK overcomes acquired resistance to cetuximab in colorectal cancer cells via induction of apoptosis. (A) Parental DiFi and cetuximab-resistant DiFi5 cells were cultured in 0.5% FBS medium without and with the indicated concentrations of cetuximab for 5 days and then subjected to MTT assay. **(B)** DiFi5 cells were cultured in 0.5% FBS medium in the presence of 20 nM cetuximab, 10 μM dorsomorphin, or both for 24 h. Cell lysates were then prepared and subjected to Western blot analysis with the indicated primary antibodies (right panel) and to a quantitative apoptosis ELISA (left panel). $^{**}p < 0.01$. **(C)** DiFi5 cells were treated with 20 nM cetuximab alone or in combination with 2.5 or 5 μM dorsomorphin for 72 h. Cell viability was determined by MTT assay. The OD values of the treated cells were normalized to the OD value of the untreated cells, which was set as 100%. Data shown are means and SDs ($n = 3$). $^{**}p < 0.01$. **(D)** DiFi5 cells were treated as described in (C). The cells were subjected to a LIVE/DEAD assay (Life Technologies) and observed under a fluorescent microscope. The ratio of dead cells to total cells in each group was calculated as described in Materials and Methods. $^{**}p < 0.01$.
